# Bone Marrow-Derived Cells in Endometrial Cancer Pathogenesis: Insights from Breast Cancer

**DOI:** 10.3390/cells11040714

**Published:** 2022-02-17

**Authors:** Alejandra I. Ferrer, Ella Einstein, Sara S. Morelli

**Affiliations:** 1 Department of Medicine, Rutgers New Jersey Medical School, Newark, NJ 07103, USA; aif25@gsbs.rutgers.edu (A.I.F.); einstein.e@northeastern.edu (E.E.); 2 School of Graduate Studies Newark, Rutgers University, Newark, NJ 07103, USA; 3 Department of Obstetrics, Gynecology and Reproductive Health, Rutgers New Jersey Medical School, Newark, NJ 07103, USA

**Keywords:** endometrial cancer, cancer stem cells, dormancy, endometrium, breast cancer, bone marrow niche

## Abstract

Endometrial cancer is the most common gynecological cancer, representing 3.5% of all new cancer cases in the United States. Abnormal stem cell-like cells, referred to as cancer stem cells (CSCs), reside in the endometrium and possess the capacity to self-renew and differentiate into cancer progenitors, leading to tumor progression. Herein we review the role of the endometrial microenvironment and sex hormone signaling in sustaining EC progenitors and potentially promoting dormancy, a cellular state characterized by cell cycle quiescence and resistance to conventional treatments. We offer perspective on mechanisms by which bone marrow-derived cells (BMDCs) within the endometrial microenvironment could promote endometrial CSC (eCSC) survival and/or dormancy. Our perspective relies on the well-established example of another sex hormone-driven cancer, breast cancer, in which the BM microenvironment plays a crucial role in acquisition of CSC phenotype and dormancy. Our previous studies demonstrate that BMDCs migrate to the endometrium and express sex hormone (estrogen and progesterone) receptors. Whether the BM is a source of eCSCs is unknown; alternatively, crosstalk between BMDCs and CSCs within the endometrial microenvironment could be an additional mechanism supporting eCSCs and tumorigenesis. Elucidating these mechanisms will provide avenues to develop novel therapeutic interventions for EC.

## 1. Introduction

Endometrial cancer is the most common gynecological cancer, representing 3.5% of all new cancer cases in the United States [1]. In recent years, there has been a 0.7% rise in EC incidence from 1999 to 2015, and 1.1% increase in associated mortality from 1999 to 2016 [2]. This rise is due to both an increase in women’s life expectancy as well as increasing incidence of obesity [3]. For cases involving localized EC, the 5-year relative survival rate is 94.9%; as compared to a rate of 69.3% in more severe cases of regional EC [1]. EC recurs in about 13% of high-risk patients and is associated with a poor prognosis [4].

EC is broadly categorized into two types, I (endometrioid) and II (non-endometrioid) [3]. Type I EC is estrogen-dependent and displays low proliferative capacity, correlating with a better prognosis and lower incidence of recurrence relative to type II [5]. Excessive estrogen exposure due to anovulation, excessive adipose tissue, and/or hormone therapies lacking progesterone, predisposes women to type I EC [3,5]. Conversely, type II EC pathogenesis is estrogen-independent, and tumors possess high metastatic potential, worsening patient prognosis and increasing cancer recurrence [6].

Irrespective of the type, EC tumors are composed of heterogenous cell subpopulations that differ in proliferative properties and sensitivity to treatment [7]. The heterogeneous tumor is composed of progenitor cancer cells and cancer stem cells (CSCs) [8]. CSCs are the source of tumor formation, leading to cancer recurrence even after long periods of remission [9]. CSCs exhibit the hallmark properties of stem cells, including the ability to self-renew and to differentiate into progenitors [8]. These cells resourcefully leverage their microenvironment to enter a dormant state, characterized by cell cycle quiescence, which confers resistance to treatment and immune evasion [9,10]. Tissue-specific CSC populations have been described, such as endometrial CSCs (eCSCs); however, a lack of consensus regarding eCSC-specific markers has hindered their isolation and characterization [4,11].

Intrinsic cues within a cancer cell are important for tumor progression; however, cancer cell survival is also dependent on their microenvironment [12]. In EC specifically, the tumor microenvironment provides support to cancer cells via contact-dependent and/or contact-independent interactions, resulting in enhanced metastatic potential [13]. In this perspective article, we will discuss the function of various cell types within the EC microenvironment and their role in promoting EC progression. Since multiple laboratories demonstrate that bone marrow-derived cells (BMDCs) take up long-term residence within the endometrium [14,15,16,17], we offer a novel perspective on how BMDCs within the tumor microenvironment may promote endometrial CSC (eCSC) survival and/or dormancy. To do this, we leverage what is known about another sex hormone-driven cancer, breast cancer, in which the BM microenvironment plays a crucial role in acquisition of CSC phenotype and dormancy [18].

## 2. Endometrial Cancer

### 2.1. Types of Endometrial Cancer

Bokhman’s dualistic model classifies EC into two pathogenic types [19]. Type I tumors are the most prevalent form of EC and are estrogen-dependent, sensitive to progestogens, and often display premalignant endometrial hyperplasia [20]. Type II tumors are estrogen-independent, arise in the endometrium, and are derived from precancerous lesions [21]. Type II ECs present earlier and more aggressively, are insensitive to progestogens, and are often resistant to standard chemotherapy and radiation [22]. Although type II tumors account for 10–20% of all cases, they account for 40% of total deaths from EC [23].Thus, type II ECs are highly malignant in comparison to type I EC, and diagnosis is associated with poor prognosis. Generally, type I ECs have endometrioid histology while most type II ECs have non-endometrioid histology such as serous carcinoma (5–10% of all ECs) and clear cell carcinoma (1–5% of all ECs) [22]. However, classification based on histology alone can be inaccurate due to overlapping of morphologies of different histologies, so molecular markers identified through immunohistochemistry are often used to aid in diagnosis.

While the Bokhman model remains valid, it fails to consider the genetic and molecular heterogeneity of EC tumors. Instead, some advocate for a classification system that incorporates both histopathological and genetic/molecular features to allow for better predictions of survival [24]. Using multiple sequencing data derived from The Cancer Genome Atlas Project (TCGA), researchers have been able to classify EC into four groups with different molecular profiles: Polymerase ε (POLE ultra-mutated), microsatellite-instable (MSI [hypermutated]), copy-number (CN) low, and CN high [25]. Depending on the tumor profile status it is possible to specifically classify different EC subtypes to improve treatment strategies. Combining molecular features with clinical elements used to identify the severity of the disease, such as stage and node status, will afford clinicians improved ability to determine the tumor type and specialized treatment needed.

### 2.2. Role of Estrogen in EC Pathogenesis

The ovarian steroid hormones estradiol and progesterone are predominant factors driving cyclic endometrial proliferation, differentiation, and shedding (menses) in a woman’s menstrual cycle. The menstrual cycle is composed of the follicular, ovulatory, and luteal phases. During the follicular phase, increasing levels of estradiol, derived from granulosa cells of ovarian follicles, stimulate endometrial epithelial and stromal cell proliferation. A mid-cycle surge in luteinizing hormone (LH) and follicle-stimulating hormone from the pituitary stimulates ovulation. Following ovulation, the luteal phase commences; during this time, the endometrium undergoes differentiation under the influence of progesterone and other steroid and peptide hormones secreted by the corpus luteum [26]. In the absence of pregnancy, circulating estradiol and progesterone levels fall in the late luteal phase, and menstrual shedding of the functional endometrial layer ensues. In the anovulatory woman (in which there is no cyclic luteal source of progesterone), prolonged periods of unopposed estrogen results in overgrowth of the endometrium which may lead to malignant transformation [27].

### 2.3. Role of Obesity in EC Pathogenesis

EC rates in high income countries such as the United States have increased in a similar manner as obesity rates over the past few decades [28]. EC incidence is directly linked to obesity; an increase in 5 BMI units augments one’s risk of EC by 50% [29]. Most EC is related to obesity due to the conversion of androgens to estrogen by aromatase within adipose fat cells, thus creating an overabundance of unopposed circulating estrogen. Opposing estrogen with administration of progestins is one strategy for treating certain type I ECs in women desiring pregnancy [29]. In fact, patients with stage IA endometroid tumors (low-grade progesterone receptor-positive) who are interested in bearing children have been shown to have excellent clinical outcomes, including pregnancy, when treated with progestins [30].

### 2.4. Role of p53 and PTEN Mutations in EC

Mutations within the TP53 (p53) gene are characteristic of EC tumors. The frequency of p53 mutations for type I and type II EC is 90% and 10–40%, respectively [31]. Missense p53 mutations are the most prevalent in EC tumors, correlating with poor patient outcomes [32]. One study demonstrated that p53 deletion causes the development of multiple type II EC subtypes that possess high metastatic potential, in vivo [33]. Additionally, EC tumors in which p53 is overexpressed display higher resistance to treatment in comparison to those without p53 mutations [34]. Higher incidence of type I EC recurrence at primary and/or secondary sites has been observed in patients with tumors that co-express p53 and estrogen receptor (ER)β proteins [35]. However, future studies addressing the potential interplay between p53 and ERβ in EC pathogenesis need to be performed.

Mutations in the phosphatase and tensin homolog (PTEN) gene, a tumor suppressor gene that controls cell proliferation [36], are also prevalent in EC tumors, encountered in approximately 37–60% of type I EC [37]. PTEN mutations are mostly present in short coding mononucleotide repeats which correlate with microsatellite instability [38]. One study showed that 61% of type I EC tumors exhibit loss of PTEN expression [39], which is associated with EC recurrence [40]. Despite this, loss of PTEN sensitizes EC tumors to PARP/PI3K inhibitors, a useful strategy to treat EC patients with metastatic disease [41]. In women older than 60 years, co-expression of PTEN and p53 in EC tumors correlates with high metastatic potential and EC recurrence [36]. Studies are ongoing to identify appropriate molecular markers for use as clinical prognostic indicators and to better guide therapy in women with EC.

## 3. Cancer Stem Cells

Tumor heterogeneity is one feature displayed by cancerous tissues that permits adaptation to different niches and microenvironmental cues. Within the tumor microenvironment, cancer stem cells (CSCs) exhibit abnormal stem cell-like behavior and are responsible for tumor repopulation and cancer resurgence [42]. CSCs undergo asymmetric division, a process that can be modulated by microenvironmental or intrinsic cues resulting in either CSC self-renewal or differentiation into cancer progenitors [43], thus contributing to the heterogeneity of the tumor. In addition, CSCs express core stem cell genes (i.e., Octamer4-a, Nanog, Sox2, and Klf4) and drug-efflux transporters (i.e., ATP-binding cassette), which serve as a protective mechanism against conventional treatments by allowing disposal of toxins from the cell [44,45]. CSCs undergo dormancy, a process characterized by cell cycle quiescence and resistance to treatment [46]. Dormancy poses a therapeutic challenge because current treatments require an active cell cycle status for successful eradication of the cells. CSCs share properties with non-malignant stem cells, complicating the development of pharmacological agents that will solely eliminate the cancer cells. Therefore, elucidating fundamental pathways and markers exclusive to CSCs is imperative to effectively target these cells without negatively impacting the non-malignant stem cells. 

CSCs were identified in solid tumors as CD44^+^/CD24^(−/low)^/Lineage^(−)^ [8,47]. Phenotypic and functional studies demonstrated the capacity of these cells in driving tumor repopulation in immunodeficient mice [47]. Further studies aimed to better stratify CSCs, leading to the identification of CD133 and aldehyde dehydrogenase-I as markers enriched in abnormal stem cell-like cells [48,49]. The identification and isolation of CSCs varies depending on the tissue of origin; thus, classification of tissue-specific markers is necessary to discern between CSCs from different sources and to better understand CSC behaviors that might be limited to specific anatomical regions [8].

The origin of CSCs is mainly attributed to two different theories. One of the theories establishes that mutations in core stem cell genes of non-malignant stem cells result in the development of CSCs [50]. Alternatively, mutations can promote the de-differentiation of progenitor cancer cells into CSCs [51]. Notably, the acquisition of an abnormal stem cell-like phenotype is a highly dynamic process that may be dictated by the cellular microenvironment. Regardless of their origin, CSCs require extrinsic (niche-driven) and intrinsic signals that allow their survival for extended periods. 

The first evidence of CSCs in the endometrium was provided by Hubbard et al. [50], demonstrating that clonally derived endometrial carcinoma cells possess a capacity for self-renewal, de-differentiation, and tumorigenic properties [50]. Identification of eCSCs poses a challenge due to a lack of consensus regarding cell-surface or intracellular markers that are solely expressed in such cells. Therefore, determining markers that are exclusive for eCSCs is necessary to better understand behaviors specific to these cells and to develop strategies that can effectively target and ameliorate disease outcomes.

## 4. Pathways Involved in eCSC Maintenance

The lack of markers specific to eCSCs has made it difficult to develop strategies to target these cells. However, a number of cell signaling pathways involved in maintaining eCSC stemness are being studied as possible therapeutic targets. A comprehensive review of established pathways that regulate eCSC maintenance is outside the scope of this review. Herein, we discuss selected pathways (i.e., Notch and Wnt signaling) and molecules (i.e., micro-RNAs) that could be implicated in EC progression driven by bone marrow-derived cells (BMDCs) recruited to the endometrium. We focus on these pathways and molecules because they play a fundamental role in the well-established model of BM-driven breast cancer dormancy. 

The Notch signaling pathway plays a major role in cell maintenance and fate [52]. Notch has been recognized as an important pathway in a multitude of solid tumor types such as breast [53], colorectal [54], and cervical [55]. This signaling cascade can induce cell proliferation, metastasis, and epithelial-to-mesenchymal transition (EMT), all of which relate to CSC maintenance [56]. EMT is a process by which cancer cells lose the polarization typically associated with epithelial cells and gain mesenchymal characteristics such as increased migration and invasiveness [57]. With respect to EC, several studies have reported that eCSCs have enhanced Notch signaling activity [4,58], and inactivation of Notch signaling reduces eCSC clonogenic capacity and resistance to treatment [58]. Downstream proteins of Notch interact with a myriad of different factors involved in other pathways to maintain CSCs and progenitors. One of these factors is Musashi-1, an RNA-binding protein known for its role in CSC maintenance [59]. Elevated levels of Musashi-1 have been observed in eCSCs and inhibition of this protein results in downregulation of Notch-1 signaling which triggers apoptosis in endometrial cancer cells (ECCs) [60].

Another pathway which has been widely studied for its role in the development of cancer and CSC maintenance is the Wnt/β-catenin pathway (Wnt pathway). In physiological conditions, this signaling pathway is associated with increased differentiation, polarization, and migration [61]. However, in cancer, Wnt signaling is crucial in maintaining CSC stemness [62]. In EC specifically, the Wnt pathway is often dysregulated, characterized by increased mutations in β-catenin and expression of Wnt ligands within endometrial tissue [63]. Three Wnt ligands (WNT7A, WNT10A, and WNT10B) are significantly elevated in EC tumors in an estrogen-dependent manner, affecting primarily type-I EC [63,64]. Wnt signaling, activated by the calcium binding protein, SPARC-related modular calcium binding 2 (SMOC-2), modulates stemness in eCSCs [65]. Interestingly, expression of SMOC-2 has been used to distinguish between eCSCs and progenitors [65].

In addition to cell signaling pathways, many micro-RNAs (miRNA) have been studied as potential therapeutic targets for EC. For the purpose of this review, we will focus on a few notable ones that have been shown to regulate eCSC functions. Overexpression of miRNA-21, for example, downregulates PTEN expression in eCSCs, resulting in cell proliferation [66]. Overexpression of miRNA-194 inhibits eCSC invasion and metastasis in vivo, by suppression of the transcription factor sex-determining region Y-box protein 3 (SOX-3), which plays a major role in EMT [67]. Certain miRNAs regulate eCSC functions by interaction with the Notch pathway. For instance, miRNA-34a has been shown to downregulate Notch-1 gene expression in ECCs, thereby inhibiting ECC proliferation, invasion, and migration [68]. Another miRNA that regulates Notch signaling is miRNA-134, which reduced eCSC proliferation and migration by downregulating eCSC expression of protein O-glucosyltransferase 1 (POGLUT) and Notch signaling [69]. Overall, several factors are key drivers of CSCs and eCSCs; thus, in-depth studies should be conducted to determine the feasibility of targeting such factors to effectively halt cancer progression. Established eCSC markers and key factors involved in eCSC maintenance are summarized in Table 1.

## 5. Endometrial Cancer Microenvironment

Inherent mutations in cancer cells are a driving force in tumor development. To prolong their survival, cancer cells leverage their niche by orchestrating either transcriptional or epigenetic changes within the stroma to allow tumor progression [12,85]. The stroma provides structural and functional support within an organ and is composed of fibroblasts, endothelial cells, epithelial, and immune cells [86]. Bidirectional communication between the stroma and cancer cells allows adaptation of the cancer cells to the niche. Herein, we will discuss the role of the stroma in EC and provide insights about molecules that support the cancer cells.

### 5.1. Cancer-Associated Fibroblasts in EC

Cancer-associated fibroblasts (CAFs) can exert a supportive role during tumor progression by enhancing proliferation, metastatic potential, and resistance to treatment in cancer cells. In EC, CAFs can promote proliferation of ECCs by upregulating PI3K/Akt and MAPK/Erk pathways [87]. Unlike normal fibroblasts, CAFs mediate proliferation of ECCs by releasing increased levels of cytokines and growth factors, such as macrophage chemoattractant protein (MCP)-1, IL-6, IL-8, RANTES and vascular endothelial growth factor (VEGF) [88]. Interestingly, higher levels of epidermal growth factor, transforming growth factor-β, hepatic growth factor, and fibroblast growth factor in the conditioned media of CAFs induces EMT in ECCs, resulting in increased invasiveness and migratory properties [89]. Furthermore, CAFs release stromal derived factor 1-α (SDF-1α/CXCL12), a chemokine which interacts with its receptor CXCR4 expressed in ECCs and subsequently enhances ECC migration [90]. Upregulation of the CXCL12/CXCR4 axis is correlated with poor prognosis in EC patients [91]. Mechanistically, downstream signaling of CXCR4 activates PI3K/Akt pathways to support ECC survival, proliferation, and migration [90]. The CXCL12/CXCR4 axis has been shown to be crucial in metastasis to distant organs in other cancer types such as breast and ovarian cancer [92,93]. Altogether, soluble factors released by CAFs contribute to enhanced ECC migration and proliferation, ultimately resulting in tumor progression.

### 5.2. Endothelial Cells in EC

Estrogen-driven angiogenesis during each menstrual cycle is necessary for regeneration of the functional layer of the endometrium. Specifically, estrogen-induced VEGF secretion from glandular epithelial and stromal cells promote vascularization in the endometrium [94]. Increased tissue levels of VEGF are associated with poor prognosis in EC patients [95]. VEGF secretion can be induced by hypoxic conditions during cancer development, triggering expression of matrix metalloproteinases, which promote neovascularization within the tumor and metastasis [96]. For instance, epithelial membrane protein-2 (EMP2), a marker for early-stage EC, mediates upregulation of hypoxia-inducible factor 1-alpha (HIF-1α) by stimulating expression of VEGF and thereby increasing capillary formation [97,98]. Indeed, in early-stage EC, patients exhibit high levels of circulating endothelial cells in comparison to healthy individuals, suggesting that angiogenesis is fundamental during development of the disease [99]. Microarray studies performed on EC-associated endothelial cells revealed enhanced microtube formation, increased invasiveness, and upregulation of ECM proteins to facilitate interaction with ECCs [100].

As noted earlier, prolonged exposure of the endometrium to estrogen, without the opposing role of progesterone, can cause type I EC [3]. In EC, estradiol induces VEGF and basic fibroblast growth factor (b-FGF) in ECCs, leading to Akt activation and downstream Nfκ-B signaling, resulting in increased tumor burden [101]. Additionally, estrogen-mediated Nfκ-B activation can be caused by ECC-secreted platelet-activating factor, facilitating vasculature sprouting [102]. Collectively, during EC development, elevated amounts of angiogenic factors are released to promote neovascularization, contributing to tumor survival.

### 5.3. Immune Cells in EC

In steady-state conditions, the immune system recognizes and eliminates cancer cells via pro-inflammatory mechanisms. Despite this, cancer cells can circumvent inflammatory responses and effectively leverage the immune system [103]. In some cases, cancer cells bias the immune system towards an anti-inflammatory response, resulting in prolonged survival, and enhanced metastatic potential [104].

In EC, innate and adaptive immune cells infiltrate the tumor, exerting either pro- or anti-tumorigenic effects. Migration of macrophages to the tumor microenvironment is critical during disease progression. EC promotes polarization of macrophages from an anti-tumor (M1) to a tumor-enhancing (M2) phenotype [105,106]. Both natural killer cells and cytotoxic CD8^+^ T cells can exert anti-tumorigenic effects, leading to decreased tumor burden. Despite this, ECCs take advantage of NK cells by promoting transcriptional changes resulting in reduced cytotoxicity and degranulation [107]. In addition, ECCs reduce recruitment of CD8^+^ T cells and release immunosuppressive cytokines to persist within the niche [108]. Ultimately, ECCs take advantage of the immune system to successfully evade conventional treatments.

## 6. Bone Marrow Niche in Hormone-Driven Cancers—Using Breast Cancer (BC) as a Model

In the bone marrow, metastatic cancer cells of certain solid tumors (e.g., breast) are known to survive in a dormant state as CSCs, later resurging as increasingly aggressive, metastatic disease [109]. Although EC rarely metastasizes to the BM, BMDCs have been shown to be recruited to the uterus [14,15]; whether these cells play a role in EC survival and/or recurrence at the primary site is unknown. Extensive studies in breast cancer have elucidated mechanisms by which cancer cells leverage the BM for their survival. In this section, we will discuss how the BM niche homes metastatic BC, and how breast cancer cells (BCCs) utilize the BM microenvironment to ensure their survival. Understanding these mechanisms provides important insights into how BMDCs recruited to the endometrium might be supporting EC at the primary site.

### 6.1. Concept of BC Dormancy

BC is the most common type of cancer among women, and like type 1 EC, is a sex hormone-driven disease. Despite recent advancements in treatment and early intervention, BC remains a clinical challenge, primarily due to increased incidence of recurrence over the years [110]. Upon recurrence, BCCs are highly metastatic and more aggressive, contributing to reduced overall patient survival [111]. However, the mechanisms underlying BC recurrence remain poorly understood. Metastatic BCCs preferentially migrate to the BM, residing in the BM for extended periods by successfully evading treatments and immune surveillance [109]. BCCs thrive in the BM microenvironment by acquiring a dormant phenotype and undergoing de-differentiation into CSCs [112,113]. Certainly, the heterogeneity of the BM provides a beneficial microenvironment for BCC survival, impeding efficacious targeting of the malignant cells. Therefore, it is important to understand how BM-niche cells support dormancy acquisition to develop strategies that can eradicate BCCs.

### 6.2. Role of BM Niche in BC Dormancy

#### 6.2.1. Perivascular Niche

The perivascular niche of the BM is composed of endothelial cells, mesenchymal stem cells, and nerve fibers sheathed throughout the blood vessels [114]. The sinusoids are capillaries distributed across the BM that are highly permeable and allow the release and/or entrance of HSCs and progenitors [115]. The permeability of the sinusoids permits the invasion of metastatic BCCs into the BM cavity [114]. Entrance of BCCs into BM is primarily mediated through the CXCL12-CXCR4 axis [93]. BM cells, such as mesenchymal stem cells (MSCs) and endothelial cells, release CXCL12 which allows recruitment of metastatic BCCs with increased expression of the receptor CXCR4 to the BM [93,116]. The upregulation of CXCR4 in BCCs is facilitated by the neuropeptide, tachykinin-precursor-1 [93].

The perivascular niche of the BM promotes dormancy in BCCs. In vivo imaging studies of the BM perivascular niche demonstrated that BCCs are in proximity to endothelial cells, suggesting a role of the vasculature in BC dormancy [117]. Endothelial cells induce BC dormancy by releasing thrombospodin-1, which restricts cancer cell proliferation [117]. Furthermore, BCCs previously exposed to high doses of chemotherapy tend to preferentially migrate to the BM, seeking refuge at the perivascular site [118]. Both the endothelium and MSCs within the BM provide protection against chemotherapy through enhanced integrin signaling [118].

MSCs are multipotent non-hematopoietic cells that support HSC maintenance. Our previous studies have shown that BCCs instruct MSCs in the perivascular niche to release microvesicles (i.e., exosomes) that contain a specific set of miRNA cargo that facilitate BC dormancy [112]. Exosomes, a form of contact-independent mediated interaction, are small microvesicles that transport various molecules such as lipids, proteins, and coding and non-coding RNAs, including miRNAs [119]. Exposure of MSCs to BCCs induces the expression and release of miRNAs 222/223 within MSC-derived exosomes, resulting in BC dormancy [112]. Introduction and packaging of anti-miRs 222/223 in BM-MSC-derived exosomes induced dormancy reversal [112]. In addition, MSCs, upon exposure to BCCs, secrete exosomes that promote de-differentiation of late progenitor BCCs into CSCs by enhancing the Wnt pathway [113]. Altogether, the perivascular niche is critical in supporting BC dormancy and transition into CSCs.

#### 6.2.2. Endosteal Niche

The endosteal niche of the BM is located near the bone area and is primarily composed of osteoblasts, osteoclasts, HSCs, MSCs, fibroblasts, adipocytes, and immune cells [120]. Long-term repopulating HSCs, which sustain hematopoiesis during an individual’s lifetime, reside at the endosteum. Various cell types from this region have been shown to be critical in HSC maintenance and overall homeostasis. However, in addition to supporting HSCs, the endosteal niche plays an important role in promoting CSC survival. Metastatic BCCs home to the endosteum and remain dormant in this region for extended periods. The dormant BCCs exhibit CSC properties and utilize the same resources that HSCs require to survive. Therefore, to avoid disruption of hematopoiesis, mechanisms developed to target CSCs in the BM need to take into consideration that HSCs reside in the same anatomical location.

Contact-dependent interactions such as gap junction intercellular communication (GJIC) between stromal cells and BCCs maintain dormancy of BCCs at the endosteum [121]. Specifically, connexin-43-mediated GJIC between stromal cells and BCCs allows the transmission of specific miRNAs that reduce proliferation of the malignant cell [121]. Since Cx43 is also expressed on HSCs, and these cells use it as a form of communication with neighboring cells, Cx43 is not a potential target for treatment of BC. Thus, identifying factors that might be facilitating communication between BCCs and BM-niche cells is imperative to target the malignant cells. For instance, we identified that N-cadherin interacts with Cx43 and promotes GJIC between CSCs and stromal cells, maintaining BC dormancy [122]. Disruption of the interaction between N-cadherin and Cx43 resulted in reversal of dormancy in BCCs [122]. In conclusion, the endosteal niche supports homing and maintenance of CSCs in the BM.

## 7. Perspective: BM-Derived Cells in EC Progression—Insights from BC

Although the general prognosis for EC patients is favorable due to early detection and intervention, approximately 13% of ECs recur, contributing to reduced overall patient survival [123]. The mechanisms accounting for the aggressiveness of EC upon recurrence remain to be elucidated. In this section, we provide insights into the mechanisms by which BMDCs recruited to the endometrium might be supporting ECC survival at the primary site, using BM niche-driven breast cancer dormancy as a model.

### 7.1. Role of CXCL12-CXCR4 Axis in BMDC Recruitment/Parallels with BC

Metastatic EC rarely migrates to the BM [124]. However, multiple studies indicate that BMDC populations migrate to the endometrium and take residence within the tissue [16,17]. Mechanisms regulating BMDC recruitment to the endometrium remain poorly understood but appear to involve inflammatory cues [125,126]. For instance, endometrial stem cells produce high levels of the chemokine CXCL12 in response to estradiol [125]. Enrichment of CXCL12 results in upregulation of its receptor CXCR4 in BMDCs, facilitating their recruitment to the endometrium [125]. In a murine model recapitulating endometrial injury, administration of BMDCs with CXCL12 enhanced cell migration to the endometrium and promoted tissue regeneration [126].

Although studies support a role for the CXCL12-CXCR4 axis in promoting recruitment of BMDCs to the endometrium, whether this pathway is implicated in migration of BMDCs during EC progression remains to be elucidated. As noted earlier, in BC, the CXCL12-CXCR4 axis promotes the recruitment of BCCs to the BM; the BM niche facilitates acquisition of a dormant phenotype and de-differentiation of BCCs into CSCs [113]. Whether this same phenomenon is occurring in EC is unknown. It is possible that in EC, BMDCs enter the endometrium via the CXCL12-CXCR4 axis and are potentially supporting eCSCs and progenitors.

### 7.2. Role of Specific BMDC Population: BM-MSC

The role of BMDCs once recruited to the endometrium is not completely understood but may either involve transdifferentiation into mature endometrial cell types and/or endometrial regeneration via paracrine factors [14,16]. In physiologic conditions, MSCs recruited from the BM take up residence within the endometrial basalis and serve as precursors for endometrial MSCs (eMSCs) which contribute to regeneration of the functionalis layer during the menstrual cycle [127]. It is possible that in EC, eMSCs of BM-MSC origin may support EC dormancy in a similar manner to BM-MSCs which promote BC dormancy [112].

To expand on this concept: transcriptome studies indicate that eMSCs express high levels of genes involved in angiogenesis, steroid hormone response, and immunomodulation [128], processes involved in EC development. Angiogenesis is critical for tumor development and progression. In the endometrium, eMSCs release exosomes that are endocytosed by endothelial cells, resulting in increased proliferation, migration, and angiogenesis in vitro [129]. Endometriosis, although a non-cancerous disease, has interesting parallels with cancer biology. For instance, eMSCs derived from patients with endometriosis promote multiple properties also implicated in cancer development/tumorigenesis, such as angiogenesis, remodeling of the extracellular matrix, and development of fibrosis [130]. Therefore, mechanistically, it is plausible that BM-MSCs recruited to the endometrium are precursors of endometrial MSCs, which in turn modulate EC by promoting angiogenesis in the tumor microenvironment.

### 7.3. Role of BM-MSC Exosomes in Promoting Dormancy

Contact-independent interactions between BM-derived cell types and ECCs may be considered as another possible mechanism mediating survival of cancer cells at the endometrial niche. As previously mentioned, BM-MSC-derived exosomes contain miRNAs that are sufficient to promote BC dormancy [112].Therefore, in EC, it is plausible that BMDCs recruited to the endometrium release exosomes containing miRNAs that similarly regulate key molecules involved in dormancy, stemness, and cell cycle progression. Indeed, although not a cancer model, during endometrial damage, BM-MSC-derived exosomes have been shown to reduce fibrotic lesions and increase the number of glands via the TGF-β1 pathway [131]. In addition, BM-MSCs upregulate the expression of miRNAs, including miR-340, in endometrial cells, resulting in endometrial regeneration following an injury [132]. Based on the findings in BC and in non-cancerous diseases of the endometrium, contact-independent signaling between BMDC and ECC (e.g., via exosomes) could be considered as a potential mechanism involved in regulation of EC. 

### 7.4. Role of Sex Hormones

Certain endometrial cancers (e.g., type I EC) are highly responsive to estrogen. However, whether BMDCs recruited to the endometrium are sensitive to sex hormones and/or support endometrial cancer via steroid hormone receptor signaling is unknown. Our studies demonstrate that BMDCs taking residence within the murine endometrium express estrogen receptor (ER) α and β, and progesterone receptor (PR), but whether these cells are steroid hormone-responsive is unclear [133,134,135]. Some cell types within the endometrium (for example, endometrial stem cells) are estrogen-responsive via paracrine signaling from neighboring cells, despite low level expression of ERα and ERβ [136]. It is unknown whether endometrial cancer stem cells in estrogen-dependent EC types express steroid hormone receptors. The fact that BMDCs home to the endometrium and express sex-hormone receptors raises the possibility of an indirect mechanism regulating eCSCs after sex-hormone stimulation.

### 7.5. Pathways Regulating Endometrial CSC Self Renewal/Maintenance: Parallels with BC

Although a multitude of pathways regulate CSC self-renewal and maintenance, a potential mechanism by which BMDCs may support ECCs is through the Wnt signaling pathway. As noted earlier, the Wnt pathway is often dysregulated in EC [63]. A question that remains to be elucidated is whether recruited BMDCs could be supporting ECCs in the primary site by modulating the Wnt pathway. In BC, it has been shown that BM-MSCs release exosomes that induce the de-differentiation of progenitor cancer cells into CSCs via activation of the Wnt pathway, allowing them to persist in the BM for extensive periods [113]. Given that BMDCs are recruited to the endometrium, we hypothesize that intercellular communication between BMDCs and ECCs may similarly play a role in regulation of pathways (i.e., Wnt signaling) that are implicated in stemness, cell cycle progression, and resistance to treatment. However, studies need to be conducted to determine whether BMDCs facilitate ECC de-differentiation (and if so, which BM-derived cell type[s]) and to mechanistically determine whether the Wnt pathway is driving this process. 

Another candidate pathway by which BMDCs may regulate EC is via the Notch signaling cascade. In physiological conditions, Notch signaling is important in cell-fate transitions, development, differentiation, and proliferation [52]. In the endometrium, during steady-state conditions, both epithelial and stromal compartments express Notch receptors, whereas the ligands are mostly expressed by epithelial cells [137]. During cancer development, activation of Notch signaling contributes to CSC self-renewal, cancer cell proliferation, resistance to treatment, and neovascularization [138]. As previously mentioned, eCSCs have enhanced Notch signaling activity [4,58]; inactivation of Notch signaling reduces eCSC clonogenic capacity and resistance to treatment [58]. Notch signaling is thus crucial in the regulation of eCSCs, but whether BMDCs support EC progression by modulating the Notch pathway remains unknown. In BC, microenvironmental cues regulate the Notch pathway resulting in cancer progression. More specifically, stroma-derived exosomes activate Notch signaling in BCCs via the NOTCH3 receptor, promoting increased proliferation and resistance to treatment [139]. In addition, BM endosteal niche cells facilitate and support BC dormancy in a NOTCH2-dependent manner [140]. Altogether, both Notch and Wnt pathways are potential mechanisms by which BMDCs could be regulating ECC at the primary site, accounting for progression and aggressiveness of the disease. These signaling pathways should be explored in mechanistic studies to determine their suitability for development of novel therapeutic strategies.

## 8. Concluding Remarks

**Figure 1:**
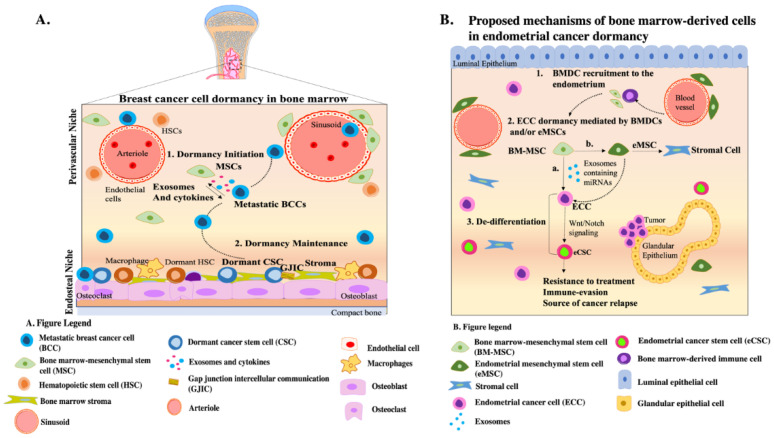
Proposed mechanisms by which BMDCs promote endometrial cancer cell (ECC) dormancy: Insights from breast cancer (**A**) (1) Breast cancer cells (BCCs) metastasize to the bone marrow (BM) and undergo dormancy acquisition facilitated by secretome exchange with perivascular mesenchymal stem cells (MSCs). (2) Dormant BCCs exhibit properties of cancer stem cells (CSCs) and establish residence at the endosteal niche where they interact with stromal cells via gap junction intercellular communication (GJIC), resulting in dormancy maintenance. (**B**) (1) BMDCs, including BM-MSCs, are recruited to the endometrium to potentially initiate ECC dormancy. (2) Mechanistically, we propose that BM-MSCs may support EC dormancy in two ways. First, (a) BM-MSCs release exosomes containing miRNAs that may initiate dormancy and (3) de-differentiation of ECCs by regulating Wnt/Notch signaling. Another mechanism may be via (b) BM-MSC differentiation into eMSC which, in turn, release exosomes that facilitate ECC dormancy and (3) de-differentiation into CSCs. Ultimately, de-differentiation of ECCs into CSCs results in resistance to treatment and immune evasion, allowing the tumor to persist for extended periods.

In this perspective article, we have offered insights regarding the recruitment of BMDCs to the endometrium and introduced the concept that these cells may play a role in survival and dormancy of ECCs. Detailed characterization of BMDCs is needed to better assess how different cell types homing to the endometrium may contribute to EC development. Since the role of BMDCs has been extensively studied in BC, a hormone-driven disease, we utilized this model (Figure 1A) to propose mechanisms by which BMDCs modulate EC survival at the primary site (Figure 1B). We hypothesize that intercellular communication between ECCs and the various BMDC types taking residence within the endometrium may facilitate disease progression by modulating pathways involved in cell cycle regulation, resistance to treatment, and stemness (Figure 1B). Future mechanistic studies must be performed to better understand the crosstalk between BMDCs and ECCs. Such insights will be critical for the development of targeted effective treatments for EC.

## Figures and Tables

**Table 1:** eCSC markers and pathways involved in stemness, resistance to treatment, and survival.

Name	Type	Function(s) within EC	References
CD133	pentaspan transmembrane glycoprotein	Modulation of stem cell genes, invasiveness, chemoresistance, tumorigenesis	[70,71]
CD44	transmembrane glycoprotein	Crosstalk with microenvironment, progression, poor prognosis, co-expression with CD133	[72,73,74]
CD117	type III receptor tyrosine kinase	Proliferation, aggression, independent prognostic factor	[75]
ALDH	enzyme	Drug resistance, independent prognostic factor	[76,77,78]
Notch	signaling pathway	Cell proliferation, apoptosis	[60]
Musashi-1	RNA-binding protein	Involved in Notch pathway; cell proliferation and apoptosis	[60]
Wnt/β-catenin	signaling pathway	Proliferation, migration, invasiveness, tumorigenicity	[79,80]
NANOG	homeobox transcription factor	Self-renewal	[50,81]
OCT-4	transcription factor	Self-renewal	[50,82]
SOX-2	transcription factor	Self-renewal	[50,82]
SMOC-2	protein	Reduce expression of stemness-related transcription factors, activate Wnt pathway	[83]
miRNA-21	miRNA	Cell proliferation	[84]
miRNA-194	miRNA	Inhibits EMT	[66]
miRNA-34a	miRNA	Inhibits Notch pathway	[67]
miRNA-134	miRNA	Inhibits Notch pathway	[68]
